# Recurrent Sesquiterpenes in *Siparuna guianensis* Essential Oil: Therapeutic Potential and Pharmacokinetic Insights

**DOI:** 10.1155/bmri/9131963

**Published:** 2026-05-14

**Authors:** Everton Luiz Pompeu Varela, Cristian dos Santos Pereira, Jorddy Neves da Cruz, Antônio Rafael Quadros Gomes, Oberdan Oliveira Ferreira, Eloisa Helena de Aguiar Andrade, Sandro Percário

**Affiliations:** ^1^ Oxidative Stress Research Laboratory, Federal University of Pará, Belém, Pará, Brazil, ufpa.br; ^2^ Postgraduate Program in Biodiversity and Biotechnology-BIONORTE Network, Federal University of Pará, Belém, Pará, Brazil, ufpa.br; ^3^ Laboratory of Functional and Structural Biology, Federal University of Pará, Belém, Pará, Brazil, ufpa.br; ^4^ Department of Morphophysiology and Physiological Sciences, Para State University, Belém, Pará, Brazil; ^5^ Adolpho Ducke Laboratory, Museu Paraense Emílio Goeldi, Belém, Pará, Brazil, museu-goeldi.br

**Keywords:** bibliometric analysis, essential oil, pharmacokinetics, sesquiterpenes, *Siparuna guianensis*

## Abstract

*Siparuna guianensis* is a neotropical medicinal plant traditionally used for inflammatory and infectious conditions, and its essential oil (EO) has been investigated for diverse biological activities, particularly antimicrobial, antiparasitic, insecticidal, anti‐inflammatory, and neuroprotective activities. However, the scientific evidence remains fragmented and predominantly preclinical, with limited integration between chemical recurrence, biological focus, and pharmacokinetic feasibility. This study performed a bibliometric review specifically of the literature on the EO of *S. guianensis* indexed in the Web of Science Core Collection and integrated it with an in silico pharmacokinetic assessment of recurrent major EO constituents. Bibliometric indicators were analyzed to identify publication trends, research focus, and knowledge gaps. Major compounds reported across studies were compiled, with emphasis on recurrent sesquiterpenes, and key ADME‐related parameters, including lipophilicity, aqueous solubility, Caco‐2 permeability, and intestinal absorption, were predicted using SwissADME and pkCSM. Eleven eligible articles were included, revealing a limited but thematically consistent research landscape, largely dominated by in vitro investigations conducted in South America. Sesquiterpenes such as germacrene D, bicyclogermacrene, and spathulenol were recurrently identified as major constituents, while *β*‐caryophyllene, *α*‐copaene, *α*‐humulene, *α*‐muurolene, *δ*‐cadinene, iso‐shyobunone, and epishyobunone were also reported among the relevant EO components and may represent candidate chemical markers. Taken together, these sesquiterpenes highlight the therapeutic potential of the EO, particularly regarding antimicrobial, antiparasitic, insecticidal, anti‐inflammatory, and neuroprotective applications. In silico predictions indicated predominantly lipophilic profiles, low‐to‐moderate aqueous solubility, high predicted Caco‐2 permeability, and elevated intestinal absorption, supporting pharmacokinetic feasibility while highlighting formulation‐related limitations. Overall, this work consolidates bibliometric, chemical, and pharmacokinetic evidence specifically related to the EO of *S. guianensis* and supports its rational interpretation as a source of bioactive volatile constituents, particularly recurrent sesquiterpenes with therapeutic promise, reinforcing the need for experimental pharmacokinetic validation, toxicological assessment, and in vivo efficacy studies.

## 1. Introduction

*Siparuna guianensis* Aubl. is a neotropical aromatic species belonging to the family Siparunaceae, a lineage distributed mainly in tropical America and represented in Brazil by several species of the genus *Siparuna* [1]. *S. guianensis* is one of the most frequently cited medicinal representatives of this group and is recognized in different regions by vernacular names such as “negramina,” “capitiú,” “limão‐bravo,” “limão‐do‐mato,” and “folha‐santa.” The species has broad geographic occurrence in the Neotropics, with records across Central and South America, and in Brazil, it has been reported in distinct phytogeographic domains, including the Amazon, Cerrado, Caatinga, Pantanal, and Atlantic Forest, highlighting its ecological amplitude and ethnobotanical relevance [2, 3].

From an ethnopharmacological perspective, *S. guianensis* has long been used by indigenous peoples, riverine groups, and rural communities in different parts of South and Central America. Traditional preparations are mainly made from the leaves, but the bark, fruits, and roots have also been reported, usually as infusions, decoctions, baths, compresses, or poultices [4, 5]. Reported folk uses include the management of pain, inflammatory disorders, fever, flu‐like symptoms, respiratory complaints, edema, rheumatism, gastrointestinal disturbances, migraine, and other infectious conditions [3, 6]. This traditional relevance is consistent with the growing pharmacological interest in the species, particularly in relation to its essential oil (EO) and other volatile constituents [6, 7].

The increasing scientific interest in *S. guianensis* is closely associated with its secondary metabolism, especially the volatile fraction obtained from the leaves. Studies that chemically characterize its EO generally describe extraction by hydrodistillation, frequently in a Clevenger‐type apparatus, followed by gas chromatography coupled to mass spectrometry (GC‐MS) for constituent identification [8]. In some studies, compound assignment is additionally supported by chromatographic retention data and comparison with a spectral library [9]. Available evidence indicates that the EO is rich in mono‐ and sesquiterpenes, but its composition is not fixed and may vary according to plant organ, population, collection site, seasonality, and chemotype. Among the constituents repeatedly reported as major compounds are germacrene D, bicyclogermacrene, spathulenol, *β*‐caryophyllene, *α*‐copaene, and, in some samples, *β*‐myrcene or E,E‐farnesol, have been repeatedly reported among the major components [8, 10, 11].

Medicinal plants remain an important source of bioactive compounds for the investigation of infectious, inflammatory, and parasitic conditions, particularly in regions where biodiversity and traditional knowledge intersect, especially in socially vulnerable settings where neglected diseases are shaped by poverty, environmental change, and limited access to health services [12–14]. In Brazil, malaria mortality and vulnerability remain unequally distributed, with a greater burden in the North Region and among socially vulnerable populations, reinforcing the relevance of regionally available medicinal resources as objects of scientific investigation [15]. Within this context, *S. guianensis* stands out as a medicinal species with ethnopharmacological relevance and a sesquiterpene‐rich EO, but with still fragmented evidence regarding standardization, safety, pharmacokinetics, and translational applicability. Accordingly, a structured synthesis of the literature is necessary to organize the currently available knowledge about this species and to identify consistent research gaps.

Therefore, the present study was aimed at performing a bibliometric analysis of the scientific literature specifically addressing the EO of *S. guianensis* and at complementing this mapping with an in silico evaluation of key pharmacokinetic properties of its recurrent major constituents, with emphasis on sesquiterpenes.

## 2. Methodology

The present study was developed based on transparent methodological and quantitative approaches, grounded in a recently developed framework entitled “Preliminary Guideline for Reporting Bibliometric Reviews of the Biomedical Literature (BIBLIO): A Minimum Requirement” [16]. Furthermore, additional details concerning the mapping of scientific knowledge were taken into consideration. This served to facilitate a more profound comprehension of the extensive scope of the generated data [17].

A search strategy was formulated using specific keywords related to the topic, with the objective of subsequently selecting relevant articles [18]. The search was conducted in the Web of Science Core Collection (WoS‐CC) database in February 2026, without imposing restrictions on time or language, using the keywords presented in Table 1. The WoS comprises numerous indexes that function as article filtration criteria. Among these, the Social Sciences Citation Index (SSCI), the Science Citation Index Expanded (SCIEXPANDED), the Arts and Humanities Citation Index (A&HCI), the Conference Proceedings Citation Index‐Social Science & Humanities (CPCI‐SSH), the Conference Proceedings Citation Index‐Science (CPCI‐S), the Book Citation Index Science (BKCI‐S), the Book Citation Index Social Sciences & Humanities (BKCI‐SSH), the Current Chemical Reactions (CCR‐EXPANDED), the Emerging Sources Citation Index (ESCI), and the Chemical Index (CI) are of particular interest. The utilization of these indexes was limited to their application as filtration mechanisms for the purpose of retrieving articles.

**Table 1:** Methodological search procedure for filtering articles in WoS‐CC.

Search strategy
TS = (“biological property” OR “biological properties” OR “biological activity” OR “biological activities” OR “ecological potential” OR “ecological potentials” OR “biological effect” OR “biological effects”) AND ts = (*Siparuna* OR “*Siparuna guianensis*” OR “*Siparuna guianensis* Aubl” OR negramina OR capitiu OR wild lemon).

The searches were conducted by two independent researchers (E.L.P.V. and C.S.P.). Discrepancies between articles were resolved through joint analysis, as described in the work of dos S. Pereira et al. [19].

### 2.1. Time Period

In the context of this study, no temporal constraints were imposed on the articles examined. A comprehensive search was conducted in WoS‐CC to retrieve articles, irrespective of publication period, with emphasis on studies that addressed *S. guianensis* as the object of study.

### 2.2. Eligibility Criteria

Original research articles and review articles addressing *S. guianensis* were selected. In addition, studies involving other species of the genus *Siparuna* were included, provided they presented relevant data on chemical composition and/or biological activities that contributed to the contextualization and comparison with *S. guianensis*.

No restrictions were imposed regarding the publication period or language. Book chapters, editorials, papers published in event proceedings, abstracts, and studies that did not correspond to the proposed theme or that were not directly related to the genus *Siparuna* were excluded.

### 2.3. Extraction of Bibliometric Parameters

The articles were exported from WoS‐CC as a Microsoft Excel document to obtain the following data: article title, year of publication, authors, number of citations, and keywords. The corresponding author′s study design, country, and continent were manually included in the file. The latter were represented using a map created with an online tool (https://mapchart.net/, accessed November 2025). Additionally, a graph in Excel represented the scientific journals in which each study was published.

A TXT file was generated from WoS to create a visualization network using the Visualization of Similarities Viewer (VOSviewer 1.6.16) software. The goal was to generate networks of citations, coauthorships, and co‐occurrences of keywords [20].

### 2.4. Data Analysis

In VOSviewer, authors with at least one article were entered as the unit of analysis for the coauthorship map. The association between authors was determined by the number of articles they coauthored. For the word co‐occurrence map, the keywords of authors with at least one publication were entered into the software as the unit of analysis. The results were presented through network visualization. In addition, the three‐field and journal graphs were generated using the Bibliometrix package in the R environment as a tool to visualize the relationships between the bibliographic fields of the analyzed dataset.

After analyzing the metrics, the documents were read in their entirety to extract relevant information about *S. guianensis*, its use in medicine, and other aspects. This process resulted in a detailed record of scientific knowledge about *S. guianensis.*

### 2.5. Analysis of Articles and Classification of Studies

We read the selected articles (*n* = 10) in detail. Information such as the date of publication, study design, country of the corresponding authors, and study results was extracted from the selected documents. We categorized the studies based on their design, taking into account the research conducted by Higgins and Green [21]. This classification is based on the Cochrane Collaboration Glossary, which includes randomized clinical trials, in vitro and in vivo studies, literature reviews, systematic reviews, meta‐analyses, and observational studies. The information provided by the articles on *S. guianensis* and its use were subsequently mapped. Additionally, the findings were described and discussed.

### 2.6. In Silico Analysis of Pharmacokinetic Properties

**Figure 1:**
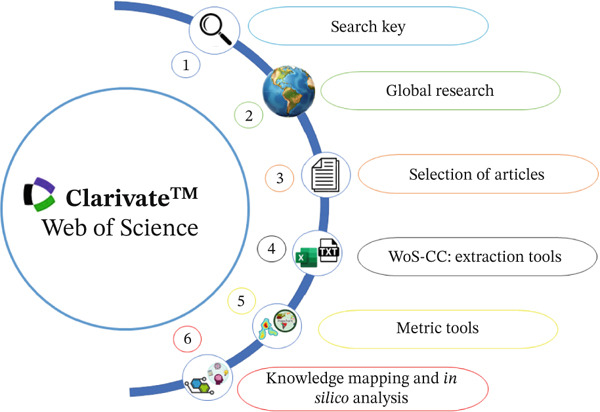
Methodological steps followed for this work.

We evaluated the pharmacokinetic properties of the major compounds present in the *S. guianensis* EO using SwissADME platforms and pkCSM [22–24]. These platforms employ approaches based on widely validated in silico prediction models to estimate absorption, distribution, metabolism, and excretion (ADME) parameters. Figure 1 shows the methodological flowchart, highlighting the steps followed in this study.

## 3. Results

The search in WoS‐CC retrieved a total of 13 articles. However, three studies were excluded for not meeting the eligibility criteria. Although they addressed species of the genus *Siparuna*, the articles “Chemical Composition and Biological Activity of the Leaf Oil of *Siparuna thecaphora* (Poepp. et Endl.) A.DC.”—DOI: “10.1080/10412905.2002.9699767” [25]; “Alkaloids From *Siparuna* (Siparunaceae) Are Predicted as the Inhibitors of Proteolysis and Plasma Coagulation Caused by Snake Venom and Potentially Counteract Phospholipase A2 Activity of Bothrops jararaca”—DOI: “10.1016/j.jep.2024.118349” [26]; and “Chemical Composition and Biological Activity of Essential Oil From Leaves and Fruits of Limoncillo (*Siparuna muricata* (Ruiz & Pav.) A.DC.)”—DOI: “10.3390/antibiotics12010082” [27] did not present relevant data for comparative contextualization with *S. guianensis*, thus disagreeing with the objective of the review.

The 10 articles total 236 citations in WoS‐CC, with an average of 23.6 citations. Table 2 details the data extracted from the selected articles to facilitate identification.

**Table 2:** Data extraction from studies selected in WoS‐CC.

Authors/year	Times cited, Wos Core	Objective	DOI
Pino‐Benitez et al., 2024 [28]	2	Determine the repellent and insecticidal activity of four EO derived from plants collected in the Chocó rainforest in Colombia against *Tribolium castaneum*.	10.37360/blacpma.24.23.4.38
Costa et al., 2022 [29]	4	Evaluate, through in silico molecular docking analyses, the potential of compounds present in essential oils from Brazilian plants, including *Siparuna guianensis*, to interact with SARS‐CoV‐2 proteins and with human host molecular targets involved in the viral infection process in order to identify possible natural candidates with antiviral activity and therapeutic potential against COVID‐19.	10.21577/0103-5053.20220043
Diniz et al., 2022 [9]	6	Chemical characterization of EO from various chemotypes of *S. guianensis* and the evaluation of their acaricidal activity against *Rhipicephalus microplus*.	10.1080/01647954.2021.2009910
Martins et al., 2021 [30]	11	Investigate the interaction between *Siparuna guianensis* Aubl. EO and the enzyme acetylcholinesterase to produce a new biologically active molecule for the treatment of Alzheimer′s disease.	10.1016/j.saa.2021.119511
Santana de Oliveira et al., 2020 [8]	52	Evaluate the chemical composition and antimicrobial activity of the primary chemical constituent in *S. guianensis* EO and simulate its interaction mechanisms using doping techniques and molecular dynamics.	10.3390/molecules25173852
de Jesus et al., 2020 [31]	17	Evaluation of the antimicrobial potential of the EO from seven native plants of the Brazilian Cerrado against foodborne disease–related bacterial strains. In addition, the chemical profile of four of these essential oils with the most promising activity is being described for the first time.	10.3390/molecules25143296
dos A. Ferreira et al., 2020 [32]	19	Evaluate the feasibility of using nanoemulsification to affect *Aedes aegypti* larvae.	10.1093/jme/tjz221
de S. Moura et al., 2020 [33]	34	The study investigated the antibacterial activity of *S. guianensis* EO against four pathogenic bacteria, assessed its toxicity in bacterial and human cells, and analyzed the mechanisms of action, including effects on cell wall permeability and molecular interactions with bacterial enzymes through in silico modeling.	10.1016/j.indcrop.2020.112142
Andrade et al., 2016 [34]	57	Evaluated the biological activity of different EOs on *Leishmania* (*L.*) *amazonensis* promastigote forms, as well as their cytotoxicity on mammalian cells and chemical composition.	10.1186/s12906-016-1401-9
Andrade et al., 2015 [35]	34	This study had analyzed the antibacterial, antifungal, and trypanocidal activities of EO from *Cinnamodendron dinisii Schwacke* (Canellaceae) and *S. guianensis* Aublet (Siparunaceae).	10.1590/S1517-838246120130683

### 3.1. Authors′ Contribution

**Figure 2:**
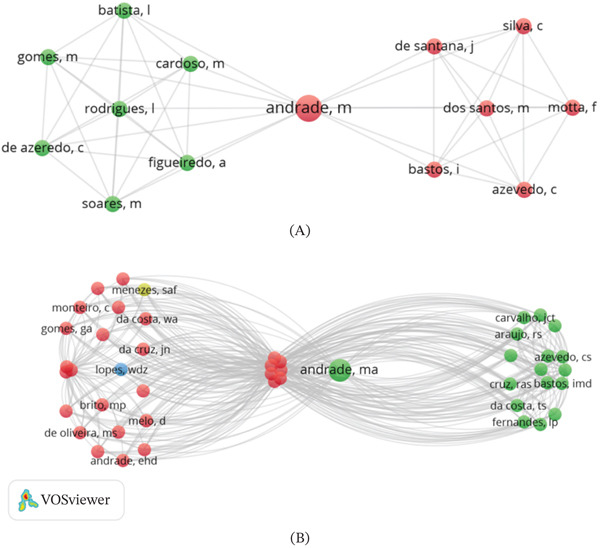
(A) Coauthorship network of researchers publishing on *Siparuna guianensis*, generated using VOSviewer. Nodes represent authors, node size is proportional to the number of publications, and links indicate coauthorship relationships, with thicker lines reflecting stronger collaborative ties. (B) Most cited authors based on the total number of citations in the Web of Science Core Collection database.

The search identified a total of 92 authors. Among these, the author with the highest number of documents was Andrade [34, 35] (*n* = 2), with a total of 91 citations. The other authors had one document each. The data presented herein indicate that, among the authors under consideration, Andrade [34, 35] exhibited the highest number of links (*n* = 66). This value indicates the level of collaboration, influence, and interconnectivity among the authors (Figure 2).

### 3.2. Country of Correspondence Authors

**Figure 3:**
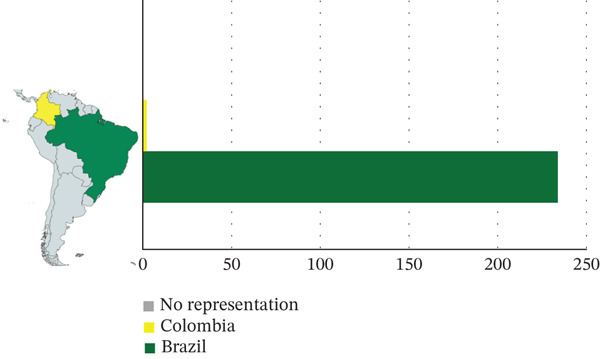
Map of South America, identifying the countries to which the correspondence authors belong, including a graphical representation of the number of documents published per country and the number of citations.

The analysis of the data revealed that Brazil was the country with the highest number of studies on *S. guianensis*, with a total of nine studies and 234 citations, followed by Colombia, with one study and two citations. As illustrated in Figure 3, all selected studies were conducted in South America.

### 3.3. Co‐Occurrence of Keywords

**Figure 4:**
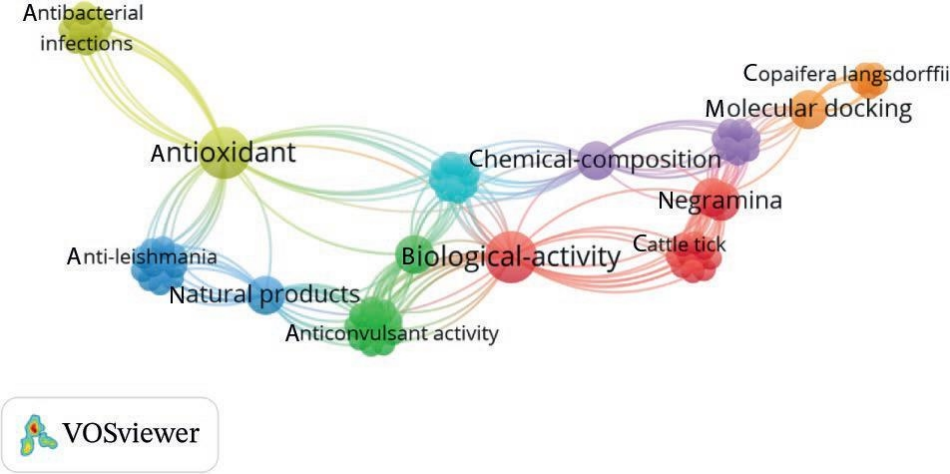
Keyword clustering analysis: The different colors separate the keywords into clusters. The thickness of the lines between them indicates the strength of the link, whereas the size of the node indicates the relevance of the topic.

A total of 105 keywords were identified. The terms with the highest co‐occurrence frequency were biological activity (*n* = 3), antioxidant (*n* = 3), natural products (*n* = 2), *cinnamodendron-dinisii* (*n* = 2), chemical‐composition (*n* = 2), negramina (*n* = 2), plant (*n* = 2), and molecular docking (*n* = 2). The remaining words occurred once each. The term with the highest total link index (*n* = 38) was “biological activity,” followed by “antioxidant” (*n* = 36), emphasizing the biological activity of *S. guianensis* (Figure 4).

### 3.4. Periodical Publications

**Figure 5:**
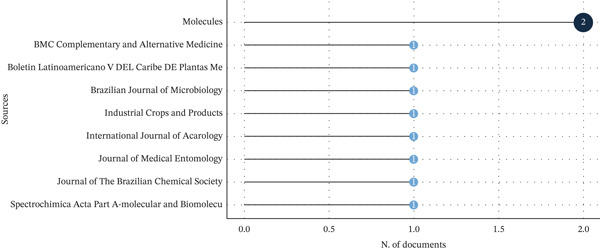
Journals in which the selected articles were published, as well as the number of citations, taking into account citations in WoS‐CC and the number of documents.

The articles selected for review were identified in nine different journals. Of these nine journals, only *Molecules* published two documents, which were cited a total of 69 times. The data presented indicate that the journals with the highest number of citations are *BMC Complementary and Alternative Medicine* (*n* = 57), *Brazilian Journal of Microbiology* (*n* = 34), and *Industrial Crops and Products* (*n* = 34) (Figure 5).

### 3.5. Classification of Study Types and Respective Years of Publication

**Figure 6:**
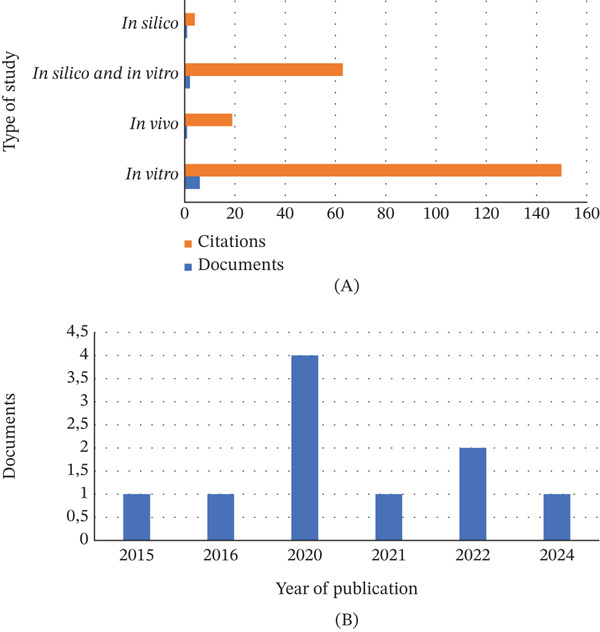
(A) The types of study, with the number of documents and number of citations. (B) The number of documents published per year.

In total, three different types of studies were identified. In vitro studies were the most common (*n* = 6), with a total of 150 citations in WoS‐CC, as shown in Figure 6. The second most frequently cited type of study was the combination of in silico and in vitro studies in the same document (*n* = 2), totaling 63 citations. In vivo and in silico studies appeared only once individually (*n* = 1), with a total of 19 and four citations, respectively. Furthermore, the aforementioned studies were published between 2015 and 2024. The year with the highest number of publications was 2020 (*n* = 4; 122 citations), followed by 2022 (*n* = 2; 10 citations). The years 2015, 2016, and 2024 had 34, 57, and two citations, respectively.

### 3.6. Knowledge Mapping

**Figure 7:**
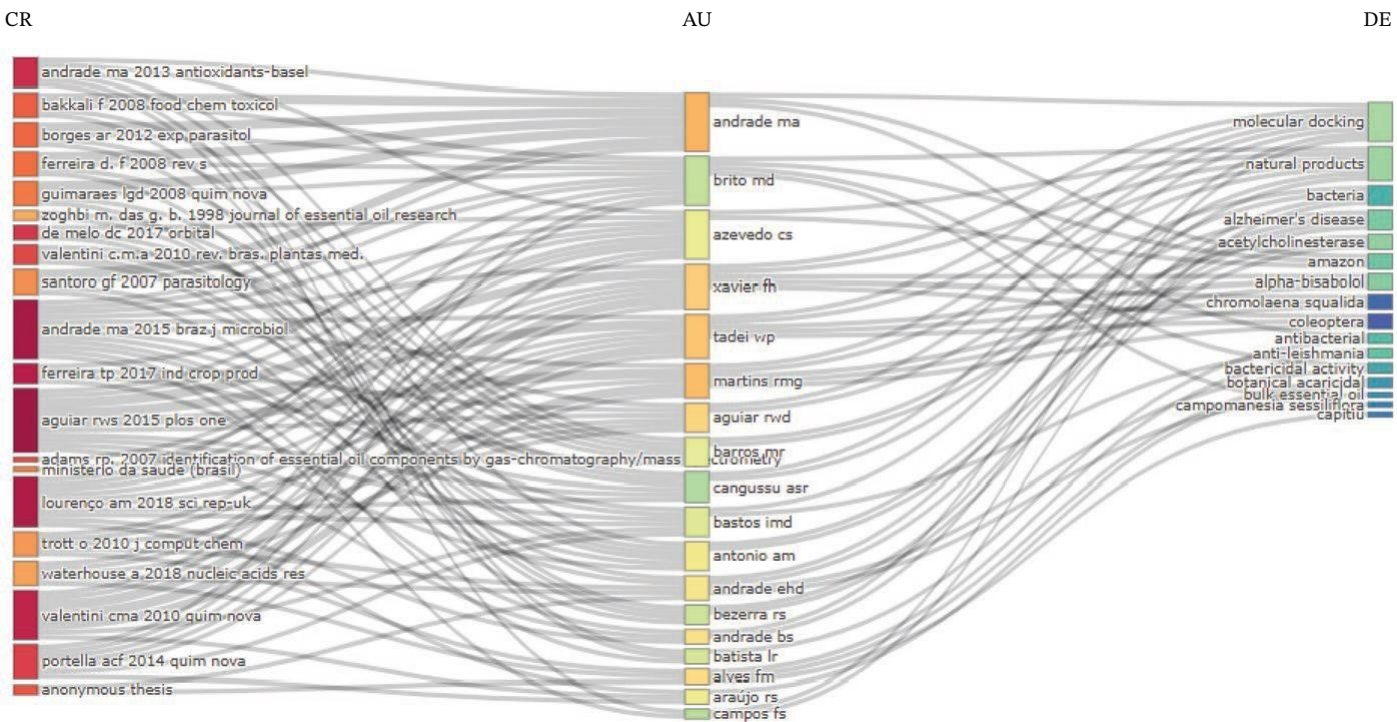
Three‐field plot graph showing the interaction of sources, authors, and descriptors. The larger the node, the more relevant a topic is, and the thicker the gray lines, the greater the connection between the categories.

Figure 7 presents a three‐axis visualization that correlates the sources (journals), authors, and descriptors utilized in the articles. This analysis facilitates the identification of the authors who published in specific journals and the primary terms employed in their research.

It is observed that authors such as Andrade M.A. and Martins M.G. have multiple connections both with previously established references and with different thematic descriptors. This intermediate position indicates that these researchers play a significant role in the articulation of scientific knowledge, integrating classic studies in the literature with new experimental and computational approaches. These authors′ connections to various references suggest that their work is grounded in well‐established theoretical foundations while simultaneously contributing to the expansion of the field of research.

Within this thematic area, the most frequently associated keywords include “natural products,” “essential oils,” “antibacterial activity,” “bacteria,” and “molecular docking,” indicating that the scientific output analyzed focuses primarily on the investigation of natural compounds and their biological activity, particularly against pathogenic microorganisms. These descriptors appear associated with different authors and references, indicating that the study of natural products with antimicrobial potential constitutes a central thematic focus of the field.

The presence of the term “molecular docking” in connection with various authors and references further suggests a growing integration between experimental approaches and computational modeling techniques, which are used to understand the molecular interactions between bioactive compounds and their potential biological targets. This connection indicates that the field of research has been incorporating tools from bioinformatics and computational chemistry to support the investigation of the mechanisms of action of natural substances.

When analyzed together, the three dimensions of the graph reveal a flow of knowledge that originates from classical references in the literature, passes through the scholarly output of key authors, and leads to specific themes represented by the descriptors. Thus, the references provide the theoretical foundation, the authors act as agents in the production and dissemination of knowledge, and the descriptors represent the thematic manifestation of the research conducted.

### 3.7. In Silico Analysis of Pharmacokinetic Properties of EO Compounds From *S. guianensis*

Table 3 presents a summary of the main metabolites identified in chemical analyses of *Siparuna* EO in different studies. The incorporation of species that are taxonomically proximate enables the contextualization of chemical variability and reinforces the significance of recurring constituents as potential chemical markers.

**Table 3:** Major constituents of essential oil from species of the genus *Siparuna.*

References	Species analyzed	Major constituents
Pino‐Benitez et al. [28]	*S. guianensis.*	*γ*‐Muurolene (13.99%) and curzerene (7.22%).
Costa et al. [29]	*S. guianensis.*	Iso‐shyobunone (23.9%), epishyobunone (18.9%), *α*‐muurolene (6.1%), t‐muurolol (5.1%), *γ*‐eudesmol (4.8%), guaiene (4.1%), spathulenol (3.7%), and elemol (3.6%).
Diniz et al. [9]	*S. guianensis*—Different chemotypes.	Chemotype 1: Germacrene D (25.13%) and bicyclogermacrene (12.49%); Chemotype 2: *β*‐Caryophyllene (21.67%) and *α*‐humulene (13.15%); Chemotype 3: Spatulenol (20.85%) and *β*‐cubebene (11.93%); Chemotype 4: *δ*‐Cadinene (24.28%) and *α*‐muurolene (13.52%).
Martins et al. [30]	*S. guianensis*.	Germacrene D (28.78%), bicyclogermacrene (13.51%), spatulenol (11.38%), and *β*‐caryophyllene (9.97%).
Santana de Oliveira et al. [8]	*S. guianensis*.	Germacrene D (36.15%), bicyclogermacrene (17.11%), spatulenol (13.85%), and *β*‐caryophyllene (8.23%).
de Jesus et al. [31]	*S. guianensis*.	Germacrene D (29.1%), spatulenol (19.2%), bicyclogermacrene (13.4%), and *α*‐copaene (9.5%).
dos A. Ferreira et al. [32]	*S. guianensis*.	Germacrene D (30.2%), bicyclogermacrene (15.6%), spatulenol (10.4%), and *β*‐caryophyllene (9.1%).
de S. Moura et al. [33]	*S. guianensis*.	Germacrene D (28.4%), bicyclogermacrene (14.8%), spatulenol (12.6%), and *α*‐copaene (9.3%).
Andrade et al. [34]	*S. guianensis*.	Germacrene D (30.4%), bicyclogermacrene (16.7%), spatulenol (13.2%), and *α*‐copaene (8.9%).
Andrade et al. [35]	*S. guianensis*.	Germacrene D (31.5%), bicyclogermacrene (14.9%), spatulenol (11.7%), and *β*‐caryophyllene (9.2%).

EOs are generally considered to be of low toxicity [36, 37]; however, it is essential to broaden the understanding of the biological behavior of their constituents. In this context, the pharmacokinetic properties of the major compounds of EO, such as partition coefficient (log P), aqueous solubility (log S), permeability in Caco‐2 cells, and intestinal absorption, were investigated. Values were predicted using SwissADME (log P and log S) and pkCSM (Caco‐2 permeability and intestinal absorption). All results obtained can be seen in Table 4.

**Table 4:** In silico pharmacokinetic properties of the major compounds of *Siparuna guianensis* essential oils.

Major compounds	Pharmacokinetic properties			
	Log P	Log S	Caco‐2 permeability	Intestinal absorption
Germacrene D	4.30	−4.03	1.43	95.59
Bicyclogermacrene	4.13	−3.72	1.41	95.01
Spathulenol	3.30	−3.17	1.49	93.90
*α*‐Copaene	3.62	−3.86	1.37	96.22
*β*‐Caryophyllene	4.23	−3.87	1.42	94.84
*α*‐Humulene	4.26	−3.97	1.42	94.68
*β*‐Cubebene	4.40	−4.01	1.39	96.97
*δ*‐Cadinene	4.14	−3.43	1.42	96.12
*α*‐Muurolene	4.07	−3.61	1.41	94.64
Safrole	2.52	−2.95	1.78	96.31
Iso‐shyobunone	2.98	−4.14	1.43	95.47
Epishyobunone	3.17	−4.06	1.32	94.65
*τ*‐Muurolol	3.18	−3.26	1.47	94.29
*γ*‐Eudesmol	3.15	−3.29	1.49	92.23
Guaiene	3.41	−3.93	1.42	95.51
Elemol	3.2	−3.8	1.51	93.48

*Note:* Log P: logarithm of the octanol–water partition coefficient, which estimates the lipophilicity of a compound. Higher values indicate greater hydrophobicity and affinity for lipid environments. Log S: logarithm of aqueous solubility (moles per liter), used to estimate the solubility of compounds in water. More negative values indicate lower solubility. Caco‐2 permeability: predicted permeability across human intestinal Caco‐2 cell monolayers, commonly expressed as log Papp (apparent permeability coefficient, centimeters per second) and used as an indicator of passive intestinal absorption potential. Intestinal absorption: predicted human intestinal absorption expressed as a percentage, representing the estimated fraction of an orally administered dose absorbed through the gastrointestinal tract.

## 4. Discussion

The present bibliometric analysis provides a structured overview of research trends, thematic focus, and knowledge gaps related to *S. guianensis*. Based on 10 eligible articles published between 2015 and 2024, the available scientific production remains limited in volume, although it shows thematic consistency, particularly regarding the biological potential of the EO. A key finding is the predominance of in vitro investigations compared to in vivo and in silico studies, which highlights an important translational limitation and reinforces the need for integrative approaches that connect chemical evidence with pharmacokinetic feasibility and safety evaluation.

Beyond publication trends, the consolidated chemical profile of *S. guianensis* EO emerges as a central element supporting its reported bioactivities. Across the analyzed studies, sesquiterpenes such as germacrene D, bicyclogermacrene, and spathulenol were recurrently identified as major constituents and were frequently associated with antimicrobial, antiparasitic, insecticidal, and anti‐inflammatory effects [6, 8, 28, 34, 38]. Additionally, other constituents, including iso‐shyobunone, epishyobunone, *α*‐muurolene, t‐muurolol, *γ*‐eudesmol, guaiene, and elemol, further reinforce the predominance of sesquiterpenes, particularly oxygenated sesquiterpenes, in the EO composition, which may contribute synergistically to its biological activities [29].

Importantly, the chemical dataset compiled in this review indicates that the EOs of *S. guianensis* are characterized not merely by the presence of terpenoids in general, but more specifically by a marked predominance of sesquiterpenes across distinct studies, collection sites, and experimental contexts. This pattern is particularly relevant because it suggests that the biological identity of the species is strongly associated with a sesquiterpene‐enriched volatile fraction, in which both hydrocarbon sesquiterpenes and oxygenated sesquiterpenes recur as dominant constituents. In this context, germacrene D, bicyclogermacrene, and spathulenol stand out as the most chemically recurrent compounds, while *β*‐caryophyllene, *α*‐copaene, *α*‐humulene, *α*‐muurolene, and *δ*‐cadinene appear as complementary markers that reinforce the sesquiterpenoid signature of the species.

From a phytochemical perspective, this recurrent sesquiterpene pattern is highly informative because sesquiterpenes are frequently associated with ecological defense, chemical communication, and adaptive responses in aromatic plants while also representing an important class of bioactive metabolites with recognized anti‐inflammatory, antimicrobial, antiparasitic, insecticidal, and neuromodulatory potential. Therefore, the repeated detection of sesquiterpenes as major constituents in *S. guianensis* supports the interpretation that these molecules are not occasional components but rather structurally and functionally relevant metabolites that may underpin a significant part of the biological activities attributed to the EO. This interpretation is strengthened by the fact that even when chemical variability is observed among chemotypes, the compositional profile remains centered on sesquiterpene dominance.

This compositional recurrence suggests that these constituents may serve as candidate chemical markers, which is relevant not only for pharmacological interpretation but also for improving standardization and reproducibility of future experimental and translational studies. Nevertheless, the chemical variability reported among different chemotypes and populations [9] represents a consistent challenge, as shifts in the relative abundance of major constituents may directly influence biological outcomes and compromise comparability between studies.

At the same time, the existence of different chemotypes should not be interpreted solely as a limitation but also as an opportunity to better understand how variations in sesquiterpene composition affect biological performance. The chemotypic shift from profiles dominated by germacrene D and bicyclogermacrene to profiles enriched in *β*‐caryophyllene/*α*‐humulene, spathulenol/*β*‐cubebene, or *δ*‐cadinene/*α*‐muurolene may have relevant implications for antimicrobial potency, acaricidal performance, anti‐inflammatory response, or target‐specific molecular interactions. Accordingly, future studies should move beyond descriptive chemical characterization and investigate whether distinct sesquiterpene‐rich chemotypes of *S. guianensis* produce reproducible differences in efficacy, selectivity, and safety.

In addition to their value as candidate chemical markers, these sesquiterpenes may also be interpreted as pharmacologically meaningful recurrence signals. In other words, the repeated identification of the same sesquiterpenes in independent studies increases the plausibility that at least part of the reported bioactivity of *S. guianensis* EO is not random but rather associated with a relatively stable set of major volatile metabolites. This is particularly relevant for germacrene D, bicyclogermacrene, and spathulenol, whose repeated predominance across the literature suggests that they should be prioritized in future studies involving bioactivity‐guided fractionation, quantitative standardization, and experimental pharmacokinetic validation.

The bibliometric mapping also revealed heterogeneous research objectives, ranging from ecological and vector‐control investigations [28, 32] to molecular modeling studies focused on neuroprotection [30] and mechanistic interactions with microbial targets [33, 37]. This diversity reflects the broad biological applicability attributed to the EO of *S. guianensis* and supports its relevance as a source of bioactive volatile constituents, particularly sesquiterpenes. However, it also underscores the lack of a consolidated translational framework capable of integrating chemical composition, mechanistic evidence, and pharmacokinetic behavior into coherent pathways for therapeutic development.

Notably, despite the diversity of investigated applications, many of these biological approaches converge toward a common chemical foundation, namely, the sesquiterpene‐rich nature of the EO. This convergence is important because it suggests that the multifunctional profile attributed to *S. guianensis* may emerge from a chemically coherent volatile matrix rather than from isolated and unrelated findings. Such coherence enhances the relevance of sesquiterpenes as central mediators of the therapeutic and biotechnological interest associated with the EO of *S. guianensis*.

The mapping reveals that the scientific field under analysis has a relatively cohesive structure, with a strong thematic focus on the bioprospecting of natural products, the evaluation of antibacterial activity, and computational molecular analysis. The interconnection between these three fields demonstrates the existence of an integrated scientific ecosystem, in which previous studies provide the foundation for new research conducted by leading authors, who, in turn, direct scientific output toward emerging topics and innovative methodologies.

Geographically, most studies have been conducted in Brazil, reflecting both the country′s biodiversity and sustained local scientific interest. Although this concentration reinforces regional research leadership, it may limit the extent to which these findings can be generalized to other neotropical regions where *S. guianensis* occurs [39]. *S. guianensis* is widely distributed across the Neotropics, with its primary occurrence in the Amazon basin, extending to other South American countries and Central America. Moreover, the species is present in additional phytogeographic domains, including the Caatinga, Cerrado, Pantanal, and Atlantic Forest, as well as in tropical Andean regions, highlighting its ecological adaptability to diverse humid and subhumid environments [3].

Despite this broad distribution, the relatively small number of indexed studies indicates that the field remains in a consolidation stage. Current scientific output is still insufficient to fully support robust translational pipelines, particularly those involving systematic safety assessment, pharmacokinetic characterization, and clinically relevant in vivo efficacy models.

An additional limitation identified in the available literature is the scarcity of data addressing toxicity, stability, pharmacokinetics, and formulation performance of *S. guianensis* EO and its major constituents. Although several biological activities have been consistently reported, the lack of standardized protocols and the limited number of in vivo studies represent major barriers to biomedical translation. In this context, the integration of bibliometric evidence with in silico pharmacokinetic prediction contributes to narrowing this gap by providing preliminary insights into the biopharmaceutical behavior of major constituents and by supporting the rational interpretation of their potential bioavailability.

The predominance of sesquiterpenes also helps explain the physicochemical behavior predicted in the present in silico analysis. Because sesquiterpenes generally exhibit higher lipophilicity than many oxygenated monoterpenes or small polar natural products, their recurrence among the major constituents of *S. guianensis* EO is consistent with the log P values observed here, as well as with the tendency toward low‐to‐moderate aqueous solubility. Thus, the sesquiterpene‐centered composition of the oil is directly connected not only to its biological promise but also to its biopharmaceutical challenges, particularly those involving solubility, formulation, and reproducibility of systemic exposure.

In this context, the major compounds exhibited predominantly lipophilic profiles, with log P values ranging from 2.52 to 4.40, which fall within a range generally considered compatible with membrane permeability and oral absorption according to classical drug‐likeness criteria [40]. However, aqueous solubility was predicted to be low to moderate (log S between −2.95 and −4.14), suggesting a potential limitation for conventional oral formulations, particularly for highly lipophilic sesquiterpenes. This physicochemical profile is consistent with EO constituents and may affect dose consistency, formulation stability, and exposure variability, which are critical parameters for therapeutic development.

Despite these solubility constraints, the predicted high Caco‐2 permeability and elevated intestinal absorption rates (> 90%) suggest that passive transcellular diffusion may represent a predominant absorption mechanism for these compounds. Importantly, this profile indicates that limited aqueous solubility may not necessarily prevent intestinal uptake but may influence the reliability of systemic exposure and the reproducibility of pharmacological effects, especially in vivo. Compounds such as safrole, which displayed a more balanced relationship between lipophilicity, solubility, and permeability, exemplify how subtle physicochemical differences may translate into distinct pharmacokinetic behaviors and highlight the importance of compound‐specific evaluation within complex mixtures.

Within this framework, the sesquiterpene‐rich profile of *S. guianensis* EO deserves special attention, since compounds such as germacrene D, bicyclogermacrene, *β*‐caryophyllene, *α*‐humulene, *δ*‐cadinene, and *α*‐muurolene combine high hydrophobicity with predicted intestinal absorption, suggesting that their in vivo performance may depend less on membrane permeation itself and more on events related to dissolution, formulation microenvironment, and metabolic fate. Oxygenated sesquiterpenes such as spathulenol, iso‐shyobunone, epishyobunone, *τ*‐muurolol, *γ*‐eudesmol, and elemol may also play an important role by modulating the overall physicochemical behavior of the oil while potentially contributing relevant pharmacological effects. Therefore, a sesquiterpene‐oriented interpretation is essential for understanding both the opportunities and the translational obstacles associated with *S. guianensis* EO.

Taken together, these findings support the interpretation that the biological potential of the EO of *S. guianensis* should be evaluated through an integrated framework combining bibliometric trends, chemical recurrence, and predicted pharmacokinetic feasibility. While in silico predictions provide valuable preliminary evidence, they do not replace experimental pharmacokinetic validation and must be interpreted cautiously, particularly in the context of complex EO mixtures and potential metabolic liabilities. Therefore, future studies should prioritize experimental pharmacokinetic profiling, standardized toxicological assessment, and in vivo efficacy models aligned with the intended biomedical applications. Additionally, formulation strategies, including nanoemulsions and other delivery systems, may be necessary to overcome solubility‐related limitations and support reproducible exposure, ultimately contributing to the rational development of *S. guianensis*‐derived therapeutic candidates.

In particular, future translational studies should prioritize the investigation of recurrent sesquiterpenes as candidate bioactive markers of *S. guianensis* EO. This includes the establishment of quantitative chromatographic parameters, the comparison of sesquiterpene‐rich chemotypes, the evaluation of possible synergistic or additive interactions among major constituents, and the development of delivery systems capable of overcoming the formulation constraints imposed by a highly lipophilic volatile matrix. Such efforts may contribute to transforming the currently descriptive knowledge about the species into a more mechanistically grounded and pharmacologically reproducible platform for therapeutic development.

## 5. Conclusion

This study provides the first bibliometric analysis specifically focused on the EO literature of *S. guianensis*, offering a structured overview of the scientific production, research trends, and knowledge gaps related to this research field. In parallel, it presents an integrated in silico ADME assessment of the major EO constituents, combining bibliometric mapping with chemical recurrence and pharmacokinetic prediction within a unified framework. Although the number of available studies remains limited, the literature shows thematic consistency, with sesquiterpenes such as germacrene D, bicyclogermacrene, and spathulenol recurrently identified as major constituents, supporting their relevance as candidate chemical markers. In silico predictions indicated predominantly lipophilic profiles, high predicted intestinal absorption, and adequate Caco‐2 permeability, while low‐to‐moderate aqueous solubility may represent a key limitation for conventional oral formulations and contribute to variability in systemic exposure. In summary, this study synthesizes scattered evidence and adds a pharmacokinetic perspective that supports the rational interpretation of the EO of *S. guianensis* as a potential source of bioactive volatile constituents, particularly recurrent sesquiterpenes. The results highlight the need for experimental pharmacokinetic validation, standardized toxicological evaluation, and clinically relevant in vivo efficacy models, as well as formulation strategies aimed at improving solubility and reproducibility. By integrating bibliometric trends, recurring chemical constituents, and predicted ADME‐related parameters, this study is aimed at providing a more organized framework for interpreting current evidence and guiding future experimental and translational research.

## Author Contributions

Conceptualization: E.L.P.V. and C.S.P. Data curation: E.L.P.V. and C.S.P. Formal analysis: E.L.P.V. and C.S.P. Funding acquisition: S.P. and E.H.A.A. Investigation: E.L.P.V. and C.S.P. Methodology: E.L.P.V. and C.S.P. Project administration: S.P. and E.H.A.A. Supervision: S.P. and E.H.A.A. Validation: S.P. and E.H.A.A. Writing—original draft: E.L.P.V., C.S.P., and J.N.C. Writing—review and editing: A.R.Q.G., O.O.F., S.P., and E.H.A.A.

## Funding

This study was partially supported by the Coordenação de Aperfeiçoamento de Pessoal de Nível Superior (CAPES), Brazil, through the Institutional Postdoctoral Program (PIPD) (Process No. 88887.030250/2024‐00).

## Conflicts of Interest

The authors declare no conflicts of interest.

## Data Availability

All relevant data are included in the manuscript.

## References

[1] Brunassi G. R. and de Lírio E. J. , Flora of Ceará: Siparunaceae, Rodriguésia. (2025) 76, 10.1590/2175-7860202576003.

[2] Rodrigues E. S. , Moreira R. D. , da Silva Ramos R. , de Souza S. A. , Sotero Filho J. W. , da Silva B. J. , Jumbo L. O. , de Oliveira E. E. , Lima E. S. , and de Souza Aguiar R. W. , Safety Assessment of the Ethanolic Extract of Siparuna guianensis: Cell Viability, Molecular Risk Predictions and Toxicity Risk for Acute and Sub-Chronic Oral Ingestion, Journal of Ethnopharmacology. (2025) 347, 119751, 10.1016/j.jep.2025.119751, 40194642. 40194642

[3] Santos D. B. , de Figueiredo R. O. , Mourão R. H. V. , Setzer W. N. , da Silva J. K. R. , and Figueiredo P. L. B. , Intraspecific Chemical Variability and Antioxidant Capacity of Siparuna guianensis Aubl Essential Oil From Brazil, Horticulturae. (2024) 10, no. 7, 10.3390/horticulturae10070690.

[4] Thomas E. , Semo L. , Morales M. , Noza Z. , Nuñez H. , Cayuba A. , Noza M. , Humaday N. , Vaya J. , and Van Damme P. , Ethnomedicinal Practices and Medicinal Plant Knowledge of the Yuracarés and Trinitarios From Indigenous Territory and National Park Isiboro-Sécure, Bolivian Amazon, Journal of Ethnopharmacology. (2011) 133, no. 1, 153–163, 10.1016/j.jep.2010.09.017, 2-s2.0-78650679092, 20888406. 20888406

[5] Odonne G. , Musset L. , Cropet C. , Philogene B. , Gaillet M. , Tareau M.-A. , Douine M. , Michaud C. , Davy D. , Epelboin L. , Lazrek Y. , Brousse P. , Travers P. , Djossou F. , and Mosnier E. , When Local Phytotherapies Meet Biomedicine. Cross-Sectional Study of Knowledge and Intercultural Practices Against Malaria in Eastern French Guiana, Journal of Ethnopharmacology. (2021) 279, 114384, 10.1016/j.jep.2021.114384, 34217796. 34217796

[6] Conegundes J. L. , da Silva J. M. , de Freitas Mendes R. , Fernandes M. F. , Pinto N. D. , de Almeida M. A. , Dib P. R. , de Oliveira Andrade R. , Rodrigues M. N. , Castañon M. C. , and Macedo G. C. , Anti-Inflammatory and Antinociceptive Activity of Siparuna guianensis Aublet, an Amazonian Plant Traditionally Used by Indigenous Communities, Journal of Ethnopharmacology. (2021) 265, 113344, 10.1016/j.jep.2020.113344, 32890711. 32890711

[7] Carvalho V. F. , Ramos L. D. , Da Silva C. A. , Nebo L. , Moraes D. , Da Silva F. F. , Da Costa N. C. , Junior R. D. , De Souza L. F. , and Rodrigues R. M. , In Vitro Anthelmintic Activity ofSiparuna guianensisextract and Essential Oil againstStrongyloides venezuelensis, Journal of Ethnopharmacology. (2020) 94, e50, 10.1017/S0022149X19000282. 30973122

[8] Santana de Oliveira M. , da Cruz J. N. , Almeida da Costa W. , Silva S. G. , Brito M. D. , de Menezes S. A. , de Jesus Chaves Neto A. M. , de Aguiar Andrade E. H. , and de Carvalho Junior R. N. , Chemical Composition, Antimicrobial Properties of Siparuna guianensis Essential Oil and a Molecular Docking and Dynamics Molecular Study of Its Major Chemical Constituent, Molecules. (2020) 25, no. 17, 10.3390/molecules25173852, 32854178. PMC7503653 32854178

[9] Diniz J. A. , Marchesini P. , Zeringóta V. , Matos R. D. , Novato T. P. , Melo D. , Vale L. , Lopes W. D. , Gomes G. A. , and Monteiro C. , Chemical Composition of Essential Oils of differentSiparuna guianensischemotypes and Their Acaricidal Activity againstRhipicephalus microplus(Acari: Ixodidae): Influence of *α*-Bisabolol, International Journal of Acarology. (2022) 48, no. 1, 36–42, 10.1080/01647954.2021.2009910.

[10] Melo D. , Miranda M. , Junior W. , Alcoba A. , Andrade P. , Silva T. D. S. , Cazal C. , and Martins C. , Anticariogenic and Antimycobacterial Activities of the Essential Oil of Siparuna guianensis Aublet (Siparunaceae), Orbital: The Electronic Journal of Chemistry. (2017) 9, no. 1, 10.17807/orbital.v0i0.930, 2-s2.0-85021254057.

[11] Lourenço A. M. , Haddi K. , Ribeiro B. M. , Corrêia R. F. T. , Tomé H. V. V. , Santos-Amaya O. , Pereira E. J. G. , Guedes R. N. C. , Santos G. R. , Oliveira E. E. , and Aguiar R. W. S. , Essential Oil of Siparuna guianensis as an Alternative Tool for Improved Lepidopteran Control and Resistance Management Practices, Scientific Reports. (2018) 8, no. 1, 10.1038/s41598-018-25721-0, 2-s2.0-85046849254, 29740112. PMC5940754 29740112

[12] Magalhães A. R. , Codeço C. T. , Svenning J.-C. , Escobar L. E. , Van de Vuurst P. , and Gonçalves-Souza T. , Neglected Tropical Diseases Risk Correlates With Poverty and Early Ecosystem Destruction, Infectious Diseases of Poverty. (2023) 12, no. 1, 10.1186/s40249-023-01084-1, 37038199. PMC10084676 37038199

[13] Alqassim A. Y. , Social, Behavioral and Environmental Determinants of Vector-Borne Diseases: A Narrative Review of Evidence and Implications for Integrated Control Approaches, Journal of Vector Borne Diseases. (2024) 61, no. 4, 525–535, 10.4103/JVBD.jvbd_34_24, 38634370. 38634370

[14] Igreja R. P. , de Macedo P. M. , and Schneider M. C. , One Health and Neglected Zoonotic Diseases, Pathogens. (2025) 14, no. 5, 10.3390/pathogens14050482, 40430802. PMC12115347 40430802

[15] Farias M. F. , Figueiredo E. R. , Silva R. N. , Galhardo D. D. , da Silva C. L. , Moreira E. M. , Azevedo Y. S. , Furtado E. C. , Castelhano J. R. , Melo-Neto J. S. , and Gomes F. D. , Malaria Mortality in Brazil: Age–Period–Cohort Effects, Sociodemographic Factors, and Sustainable Development Indicators, Tropical Medicine and Infectious Disease. (2025) 10, no. 2, 10.3390/tropicalmed10020041, 39998045. PMC11860777 39998045

[16] Montazeri A. , Mohammadi S. , Hesari P. M. , Ghaemi M. , Riazi H. , and Sheikhi-Mobarakeh Z. , Preliminary Guideline for Reporting Bibliometric Reviews of the Biomedical Literature (BIBLIO): A Minimum Requirements, Systematic Reviews. (2023) 12, no. 1, 10.1186/s13643-023-02410-2, 38102710. PMC10722750 38102710

[17] Donthu N. , Kumar S. , Mukherjee D. , Pandey N. , and Lim W. M. , How to Conduct a Bibliometric Analysis: An Overview and Guidelines, Journal of Business Research. (2021) 133, 285–296, 10.1016/j.jbusres.2021.04.070.

[18] Bakkalbasi N. , Bauer K. , Glover J. , and Wang L. , Three Options for Citation Tracking: Google Scholar, Scopus and Web of Science, Biomedical Digital Libraries. (2006) 3, no. 1, 10.1186/1742-5581-3-7, 2-s2.0-33746910186, 16805916. PMC1533854 16805916

[19] Pereira C. D. , Cruz J. N. , Ferreira M. K. , Baia-da-Silva D. C. , Fontes-Junior E. A. , and Lima R. R. , Global Research Trends and Hotspots Analysis of the Scientific Production of Amitriptyline: A Bibliometric Approach, Pharmaceuticals. (2023) 16, no. 7, 10.3390/ph16071047, 37513958. PMC10386017 37513958

[20] Waltman L. , van Eck N. J. , and Noyons E. C. M. , A Unified Approach to Mapping and Clustering of Bibliometric Networks, Journal of Informetrics. (2010) 4, no. 4, 629–635, 10.1016/j.joi.2010.07.002, 2-s2.0-77956488198.

[21] Higgins J. P. and Green S. , Cochrane Handbook for Systematic Reviews of Interventions, 2008, Wiley.

[22] Pires D. E. V. , Blundell T. L. , and Ascher D. B. , pkCSM: Predicting Small-Molecule Pharmacokinetic and Toxicity Properties Using Graph-Based Signatures, Journal of Medicinal Chemistry. (2015) 58, no. 9, 4066–4072, 10.1021/acs.jmedchem.5b00104, 2-s2.0-84929377653, 25860834. 25860834 PMC4434528

[23] Daina A. , Michielin O. , and Zoete V. , SwissADME: A Free Web Tool to Evaluate Pharmacokinetics, Drug-Likeness and Medicinal Chemistry Friendliness of Small Molecules, Scientific Reports. (2017) 7, no. 1, 42717, 10.1038/srep42717, 2-s2.0-85014610206, 28256516. 28256516 PMC5335600

[24] Cascaes M. M. , Silva S. G. , Cruz J. N. , Santana de Oliveira M. , Oliveira J. , Moraes A. A. , Costa F. A. , da Costa K. S. , Diniz do Nascimento L. , and Helena de Aguiar Andrade E. , First Report on the Annona exsucca DC. Essential Oil and In Silico Identification of Potential Biological Targets of Its Major Compounds, Natural Product Research. (2022) 36, no. 15, 4009–4012, 10.1080/14786419.2021.1893724, 33678086. 33678086

[25] Vila R. , Iglesias J. , Cañigueral S. , Santana A. I. , Solís P. N. , and Gupta M. P. , Chemical Composition and Biological Activity of the Leaf Oil ofSiparuna thecaphora(Poepp. et Endl.) A.DC, Journal of Essential Oil Research. (2002) 14, no. 1, 66–67, 10.1080/10412905.2002.9699767, 2-s2.0-0036173843.

[26] Fernandes D. A. , Gomes B. A. , Mendonça S. C. , de Castro Pinheiro C. , Sanchez E. O. , Leitão S. G. , Fuly A. L. , and Leitão G. G. , Alkaloids From Siparuna (Siparunaceae) Are Predicted as the Inhibitors of Proteolysis and Plasma Coagulation Caused by Snake Venom and Potentially Counteract Phospholipase A2 Activity of Bothrops jararaca, Journal of Ethnopharmacology. (2024) 332, 118349, 10.1016/j.jep.2024.118349, 38762214. 38762214

[27] Morocho V. , Hidalgo-Tapia M. , Delgado-Loyola I. , Cartuche L. , Cumbicus N. , and Valarezo E. , Chemical Composition and Biological Activity of Essential Oil From Leaves and Fruits of Limoncillo (Siparuna muricata (Ruiz & Pav.) A. DC.), Antibiotics. (2023) 12, no. 1, 10.3390/antibiotics12010082, 36671283. PMC9855075 36671283

[28] Pino-Benitez N. , Torralbo-Cabrera Y. P. , and Stashenko E. E. , Repellent and Insecticidal Activity of Four Essential Oils From Plants Recolleted in Chocó-Colombia Against Tribolium castaneum, Boletin Latinoamericano y del Caribe de plantas Medicinales y Aromaticas. (2024) 23, no. 4, 568–576, 10.37360/blacpma.24.23.4.38.

[29] Costa R. , Martins R. , de Lima G. , Stamford T. , Tadei W. , Maciel M. A. , Rêgo A. C. , and Xavier-Júnior F. H. , Molecular Docking In Silico Analysis of Brazilian Essential Oils Against Host Targets and SARS-CoV-2 Proteins, Journal of the Brazilian Chemical Society. (2022) 33, no. 10, 1219–1235, 10.21577/0103-5053.20220043.

[30] Martins R. M. G. , Xavier-Júnior F. H. , Barros M. R. , Menezes T. M. , de Assis C. R. D. , de Melo A. C. G. R. , Veras B. O. , Ferraz V. P. , Filho A. A. M. , Yogui G. T. , Bezerra R. S. , Seabra G. M. , Neves J. L. , and Tadei W. P. , Impact on Cholinesterase-Inhibition and In Silico Investigations of Sesquiterpenoids From Amazonian Siparuna guianensis Aubl, Spectrochimica Acta Part A: Molecular and Biomolecular Spectroscopy. (2021) 252, 119511, 10.1016/j.saa.2021.119511, 33561686. 33561686

[31] de Jesus G. S. , Micheletti A. C. , Padilha R. G. , de Souza J. , de Paula F. M. , Alves C. R. B. , Leal F. R. , Garcez W. S. , and Yoshida N. C. , Antimicrobial Potential of Essential Oils From Cerrado Plants Against Multidrug−Resistant Foodborne Microorganisms, Molecules. (2020) 25, no. 14, 10.3390/molecules25143296, 32708062. PMC7397120 32708062

[32] Ferreira R. M. , D’haveloose N. P. , Cruz R. A. , Araújo R. S. , Carvalho J. C. , Rocha L. , Fernandes L. P. , Da Costa T. S. , Fernandes C. P. , and Souto R. N. , Nano-Emulsification Enhances the Larvicidal Potential of the Essential Oil of Siparuna guianensis (Laurales: Siparunaceae) Against Aedes (Stegomyia) aegypti (Diptera: Culicidae), Journal of Medical Entomology. (2020) 57, no. 3, 788–796, 10.1093/jme/tjz221, 31840745. 31840745

[33] de Souza Moura W. , de Souza S. R. , Campos F. S. , Cangussu A. S. , Santos E. M. , Andrade B. S. , Gomes C. H. , Viana K. F. , Haddi K. , Oliveira E. E. , and Nascimento V. L. , Antibacterial Activity of Siparuna guianensis Essential Oil Mediated by Impairment of Membrane Permeability and Replication of Pathogenic Bacteria, Industrial Crops and Products. (2020) 146, 112142, 10.1016/j.indcrop.2020.112142.

[34] Andrade M. A. , Azevedo C. D. , Motta F. N. , Santos M. L. , Silva C. L. , Santana J. M. , and Bastos I. M. , Essential Oils: In Vitro Activity Against Leishmania amazonensis, Cytotoxicity and Chemical Composition, BMC Complementary and Alternative Medicine. (2016) 16, no. 1, 10.1186/s12906-016-1401-9, 2-s2.0-84996911553, 27825341. PMC5101707 27825341

[35] Andrade M. A. , Cardoso M. D. , Gomes M. D. , Azeredo C. M. , Batista L. R. , Soares M. J. , Rodrigues L. M. , and Figueiredo A. C. , Biological Activity of the Essential Oils From Cinnamodendron dinisii and Siparuna guianensis, Brazilian Journal of Microbiology. (2015) 46, no. 1, 189–194, 10.1590/S1517-838246120130683, 2-s2.0-84930419636, 26221107. 26221107 PMC4512063

[36] Bezerra F. W. F. , De Oliveira M. S. , Bezerra P. N. , Cunha V. M. B. , Silva M. P. , Da Costa R. H. H. , Pinto R. M. , Cordeiro J. N. , Da Cruz A. M. J. , Neto C. , and Junior R. N. C. , Asiri I. and Isloor A. M. , Extraction of Bioactive Compounds, Green Sustainable Process for Chemical and Environmental Engineering and Science: Supercritical Carbon Dioxide as Green Solvent, 2019, Elsevier, 149–167.

[37] de Oliveira M. S. , Silva S. G. , da Cruz J. N. , Ortiz E. , da Costa W. A. , Bezerra F. W. F. , Cunha V. M. B. , Cordeiro R. M. , and Neto A. M. d. J. C. , Inamuddin Mobin R. and Asiri A. M. , Supercritical CO2 Application in Essential Oil Extraction, Industrial Applications of Green Solvents-Volume II, 2019, 1–28.

[38] Aguiar R. W. S. , dos Santos S. F. , da Silva Morgado F. , Ascencio S. D. , de Mendonça Lopes M. , Viana K. F. , Didonet J. , and Ribeiro B. M. , Insecticidal and Repellent Activity of Siparuna guianensis Aubl. (Negramina) Against Aedes aegypti and Culex quinquefasciatus, PLoS One. (2015) 10, no. 2, e0116765, 10.1371/journal.pone.0116765, 2-s2.0-84922387957, 25646797. 25646797 PMC4315403

[39] Gonçalves V. , Silva A. , Baesse C. , and Melo C. , Frugivory and Potential of Birds as Dispersers of Siparuna guianensis, Brazilian Journal of Biology. (2015) 75, no. 2, 300–304, 10.1590/1519-6984.11413, 2-s2.0-84936741839, 26132011. 26132011

[40] Lipinski C. A. , Lombardo F. , Dominy B. W. , and Feeney P. J. , Experimental and Computational Approaches to Estimate Solubility and Permeability in Drug Discovery and Development Settings, Advanced Drug Delivery Reviews. (2001) 46, no. 1–3, 3–26, 10.1016/S0169-409X(00)00129-0, 2-s2.0-0035289779, 11259830. 11259830

